# Application of Probabilistic Risk Assessment in Establishing Perchlorate and Goitrogen Risk Mitigation Strategies

**DOI:** 10.3390/ijerph120910374

**Published:** 2015-08-26

**Authors:** Douglas Crawford-Brown

**Affiliations:** Cambridge Centre for Climate Change Mitigation Research (4CMR), Department of Land Economy, University of Cambridge, 19 Silver Street, Cambridge CB3 9EP, UK; E-Mail: djc77@cam.ac.uk; Tel.: +44-1223-760550 (ext. 123)

**Keywords:** cumulative risk, aggregate risk, goitrogens, perchlorate, regulatory rationality, probabilistic risk assessment

## Abstract

This paper applies probabilistic risk assessment in quantifying risks from cumulative and aggregate risk pathways for selected goitrogens in water and food. Results show that the percentages of individuals with a Hazard Index (HI) value above 1 ranges between 30% and 50% both with and without serum half-life correction when a traditional regulatory assessment approach based on establishment of a No Observed Effects Level (NOEL) is used. When an exposure-response curve is instead used and a threshold of 50% inhibition is assumed, 1.1% or less of the population exceeds an HI value of 1 with no serum half-life correction, rising to as high as 11% when serum half-life correction is applied. If 0% to 5% threshold for iodide uptake inhibition is assumed for production of adverse effects, the percentage of the population with an HI above 1 is 46.2% or less with no serum half-life correction, and 47.2% or less when serum half-life correction is applied. The probabilistic analysis shows that while there are exposed groups for whom perchlorate exposures are the primary cause of individuals having HI values above 1, these constitute significantly less than 1% of the population. Instead, the potential risk from exposure to goitrogens is dominated by nitrates without serum half-life correction and thiocyanates with serum half-life correction, suggesting public health protection is better accomplished by a focus on these and other goitrogens expect in highly limited cases where waterborne perchlorate is at unusually high concentrations.

## 1. Introduction

There is increasing attention in the US and EU on perchlorate as a potential public health risk in both food and water [1]. That attention began with a focus on waterborne exposures, although there are equally significant exposure routes through food. Therefore, the issue of perchlorate exposure is at least one of aggregate risk assessment [2], with attention directed towards all routes of exposure to perchlorate when considering protection of the public health. Additionally, perchlorate’s mode of action is primarily through inhibition of uptake of iodide into the thyroid, largely through the sodium-iodide symporter mechanism. In this regard, perchlorate shares a mode of action with a broad class of goitrogens, or compounds that suppress the function of the thyroid gland through interfering with iodide uptake. Hence perchlorate exposures should be seen in a framework of cumulative risk assessment, where it is one of a number of goitrogens that act collectively in reducing thyroid function and producing adverse health impacts on metabolism and development.

To place perchlorate into this aggregate and cumulative risk framework, a previous paper [3] explored the implications of alternative approaches to the development of a Reference Dose (RfD as used in the US; the equivalent in the EU is the TDI or Tolerable Daily Intake, and the equivalent in the World Health Organization (WHO) is the PMTDI or Provisional Maximal Tolerable Daily Intake) and subsequent regulatory limit on exposure to perchlorate when simultaneous exposure to other goitrogens such as thiocyanates and nitrates was present. That paper used the traditional risk-based approaches to establishing public health exposure limits based on No Observed Adverse Effects Levels (NOAELs), Lowest Observed Adverse Effects Levels (LOAELs) and margins of safety embodied in uncertainty factors to account in part for inter-subject variability in exposure. In each of the approaches, the starting point was a study by Greer *et al*. [4] of the effects of perchlorate on iodide uptake inhibition in the thyroid, which has been used to establish a NOAEL and LOAEL for regulation in the U.S..

The paper [3] identified four potential approaches to establishing a limit on exposures to perchlorate in protection of public health, consistent with this traditional methodology, described there as:

- Approach 1: Use the Greer *et al.* [4] study in the procedure typically followed in regulatory risk assessment in establishing exposure limits by a single pathway to a single contaminant, where the NOAEL or LOAEL for exposure to perchlorate alone, and solely in water, is used to establish the relevant exposure limit on perchlorate in water without reference to other goitrogens with the same mode of action.
- Approach 2: Use the Greer *et al.* [4] study to produce an exposure-response relationship for iodide uptake inhibition, coupled to the current best scientific estimate of the percentage inhibition necessary to produce a down-stream adverse effect. Again, this approach assumes exposure to perchlorate only within the class of goitrogens, or at least assumes the measured effect in the Greer *et al.* [4] study is the incremental effect resulting from the intakes of perchlorate alone.
- Approach 3: Use the Greer *et al.* [4] NOEL (0.007 mg/kg-day), but incorporate the contribution of the other goitrogens to the total goitrogen intake in that study. This includes consideration that the NOEL from that study is not a NOEL associated solely with administered perchlorate during the study, but a NOEL reflecting the combined effect of all goitrogens present in the diets of the subjects.
- Approach 4: Use the Greer *et al.* [4] study to produce an exposure-response relationship for iodide uptake inhibition, coupled to an estimate of the percentage iodide uptake inhibition necessary to produce a down-stream adverse health effect. The approach includes consideration of total background goitrogen exposure in the study population. Hence, it combines approaches 2 and 3.

As shown by Crawford-Brown [3], these four approaches lead to differences in the resulting regulatory limit on exposure to perchlorate in drinking water. In addition, two approaches were identified to establishing a Perchlorate Equivalent Concentration (PEC) to reflect the combined actions of mixtures of goitrogens considered. For those PEC values, the summary of results by Tonacchera *et al.* [5] were first used, with the effectiveness of inhibition per unit serum concentration suggesting ratios of 1:8.8:150 for perchlorate, thiocyanates and nitrates, respectively, on the basis of equivalent serum concentration. The second approach corrected for circulation half-lives in serum and produced ratios of 1:0.5:240 on the basis of ingested (mass) quantity. These two sets of ratios are used in the present study as upper and lower bounding estimates for the modelling that follows.

In addition to these four approaches rooted in traditional risk-based policies for exposure to environmental risk agents, probabilistic risk assessment has emerged as a more scientifically defensible and robust process for establishing both cumulative/aggregate risk and for establishing risk management strategies [6]. That probabilistic approach, employing Monte Carlo analyses of exposures and risks, has been applied in the past in regulatory risk assessments of risk agents in water [7]. It also is applied routinely in risk assessment and management for exposures to airborne toxicants within the USEPA, developed in part (but with other influences as well) to address issues of environmental justice as population segments exist that are at consistently higher risks due to overlapping exposures to multiple risk agents and exposure routes.

The current study expands on the analysis of the first paper [3] to assess public health risks from perchlorate within the context of aggregate and cumulative risk assessment, placing that assessment into a framework of probabilistic risk assessment using Monte Carlo analysis. The central research question—which is also the basis for potential risk-based exposure limits and risk management strategies—is: When inter-subject variability in perchlorate and goitrogen levels in food and water are modelled, what is the inter-subject variability distribution of risks, and what percentage of the population is protected adequately against these risks by any specified exposure pathway and goitrogen.

The application of probabilistic risk assessment is based on recognition that reduction in a regulatory limit on exposure can be seen through any of three perspectives:

- It reduces the mean (average) risk in a population;
- It reduces the percentage of the population or subpopulation exposed to risks above some level of acceptable risk (or increases the percentage below this level of acceptable risk);
- It increases the confidence that the mean risk in a population is below this level of acceptable risk.

For the current paper, attention is focused on the second perspective. The argument is that a regulatory limit and/or risk reduction strategy on perchlorate and goitrogens ought to be protective (against unacceptable levels of risk) for at least some specified percentage of the exposed population, cognizant that there will always be some subpopulations whose sensitivity and/or susceptibility places them at extremes requiring risk management strategies beyond those afforded by traditional controls on general ambient concentrations. In traditional policy approaches, the issue of inter-subject variability of risk is dealt with through application of an uncertainty factor specifically tied to the magnitude of inter-subject variability, with a default uncertainty factor of 10 if the data are not already reflective of the more sensitive and susceptible subpopulations. That approach, however, does not allow calculation of the percentage of the population protected by the resulting regulatory limit, although there have been studies to provide probabilistic interpretations of such an assignment [8]. It is not possible under that default non-probabilistic approach to determine whether the percentage protected is reasonable, excessive or insufficient, or whether the percentage protected is consistent across different regulations for different risk agents.

This weakness of the traditional approach—of not being able to specify the percentage of the population at excessive levels of risk—is resolved through use of formal probabilistic risk assessment methods. The current paper explores the issue of regulatory limits to perchlorate and two other goitrogens through the perspective of such a probabilistic approach and providing a more scientifically robust approach to risk assessment and management in which inter-subject variability of risk is quantified and used to determine the percentage of the population whose risk is unacceptably large. This analysis further allows risk managers to determine where limited regulatory and risk mitigation resources should be applied most effectively in protection of public health.

A significant advantage of the probabilistic approach in public health protection is its ability to incorporate the concept of a “risk cup”.This concept arose initially out of concerns for environmental justice [9] where some highly exposed groups are exposed to multiple pollutants (such as multiple goitrogens) by multiple pathways (such as water and food), each of which is at an accepted regulatory limit but whose combined effect is a risk exceeding that which would normally be allowed if the combined effect of the pollutants were quantified. The risk cup is the total risk experienced by an individual when one considers both aggregate and cumulative exposures. The current study explores a sub-set of the total risk cup, namely exposures to three goitrogens.

Rather than applying regulatory limits on exposure individually to each contaminant through each route in risk management decisions, probabilistic risk assessment allows the regulatory community to identify the fraction of people exposed to unacceptably large risks in a specific, real, population; to identify why they are at such high levels of risk (what agents and routes are contributing most significantly); and to target risk reduction resources most effectively at reducing this risk in the affected subpopulations. Note that the focus here is not on a hypothetical individual exposed to all contaminants and all routes at the maximally allowed limits for each separate contaminant and route, but rather on the actual exposures of real individuals in the population so limited regulatory resources can have the greatest effect on reducing the risks in the vulnerable populations.

The current study considers the risk cup for goitrogens only, and their action through iodide uptake inhibition by the thyroid. It is therefore a limited application of the concept of the risk cup. However, it examines the risk cup in the context of the three goitrogens most often discussed in regard to risk management decisions rooted in concern for iodide uptake inhibition by the thyroid.

## 2. Methodology

Risk of adverse effect is modelled here through calculation of the inter-subject variability distribution of the Average Daily Rate of Intake (ADRI) of a risk agent. Note that this is the same set of units as the daily intake associated with safe levels of consumption (RfD, or the units of TDI or PMTDI can be used when placed onto a per-unit-body-mass basis) that underlies the traditional approaches to regulatory risk assessment. To be more specific, the units of ADRI are μg (of the risk agent) per kg (of body mass of the exposed individual) per day, expressed as μg/kg-day.

The focus here is on direct application of exposure-response relationships for the goitrogens considered, rather than development of a Benchmark Dose (BMD) followed by application of uncertainty factors. The analysis is therefore restricted to approaches 3 and 4 mentioned previously.

For a single risk agent and environmental medium or exposure route, the calculation of the ADRI is:

ADRI = C × EF
(1)

where C is the concentration in the environmental medium (µg /L for waterborne exposures; µg /kg food product for food exposures) and EF is the Exposure Factor (L of water per kg body mass per day for waterborne exposures; μg of food product per kg body mass per day for food exposures). The choice to link intake rate of an environmental medium (e.g., L of water consumed per day) and body mass into a single Exposure Factor is due to these two components being at least partially statistically correlated (people of high body mass also have higher rates of intake of water and foodstuffs).

Both C and EF exhibit significant inter-subject variability due to age and diet. The variability in C is due to two main factors:

- Concentrations in a given environmental medium fluctuate in time;
- Time-averaged concentrations in a given environmental medium vary in space and hence. between exposed populations.

The present study is concerned only with the second cause of inter-subject variability because the underlying Greer *et al.* study [4] considers medium-term exposure over several weeks, where short-term fluctuations in concentration will be averaged.

Regarding the first cause, an important caveat on the results developed here relates to the treatment of random sampling with the variability distribution for concentrations. For water, an individual might be exposed to the same source of water consistently during the exposure period of interest (unless they are travelling). Therefore, the individual would have approximately the same value of C throughout the biologically relevant period over which the concentration is to be averaged. The length of time over which averaging is to take place depends on the assumption made about the period of exposure required to initiate adverse effects. In the US, focus has been on acute intakes where averaging is less important, whereas in the EU focus has been on chronic intakes where averaging is more significant. The focus in the present study is on intakes that take place over several weeks, again due to this being the exposure period of the Greer *et al.* [4] study.

For food exposures, however, it is not the case that concentration will remain constant throughout the exposure period. The individual will instead consume different foods and from different geographic sources during the exposure period. This will have the effect of “averaging out” variations in concentration for an individual for food exposures. Unfortunately, there is insufficient data available to reflect this averaging over the biologically relevant exposure period, as it would require temporal correlations between concentrations at the level of individual diets, and so the assumption here is one of uncorrelated sampling from the variability distribution of food concentration as was the case for water. This assumption will produce an over-dispersed inter-subject variability distribution, increasing the fraction of individuals in the “tails” of the distribution and hence the fraction of people at largely elevated levels of risk. It is justified here as being health protective.

The Exposure Factor (EF) characterises the rate at which individuals take in the various environmental media through their diets. The choice to focus on rates of intake per unit body mass in the current study is both because the ADRI uses that unit (as described above) and because the dose of a risk agent to cells depends on the degree of dilution of the agent within the body, which in turn depends on body mass.

Both C and EF show inter-subject variability, and hence ADRI will show variability as well. Both quantities tend to be lognormally distributed, as is the case for many environmental and biological parameters [10]. A lognormal distribution is described in this study by a median value in the population (the value of C or EF for which 50% of the population is above and below this value) and by a geometric standard deviation (GSD), which is the equivalent of a standard deviation in a normal or Gaussian distribution. For a lognormally distributed quantity, 68% of the values (of C or EF) are in the interval between the median divided by and multiplied by the GSD.

Lognormal distributions of biological and environmental properties also tend to show truncation of the distributions at between two and three geometric standard deviations away from the median value. Truncation in this case means that numerical values of the quantity of interest (such as EF value) are not found in the sample data—or in nature—beyond two or three GSDs from the median value. This same truncation is applied in the current study with a GSD of 3 for all of the distributions sampled.

Consider a case in which there are two compounds (Compounds 1 and 2) present in exposures of an individual. Their individual ADRI values are shown as ADRI_1_ and ARDI_2_. Note that these are the actual values of the ADRI for the individuals in an exposed population, not a hypothetical value if the individuals were exposed at the regulatory limit of each exposure pathway. Hence they are a scientific and not a policy characterisation of exposures.

Their respective RfD values (one for each of the two risk agents) will be RfD_1_ and RfD_2_; again, the same methodology can be applied using the TDI or PMTDI and would yield the same quantitative results, but only an RfD-based analysis is performed here. Their HQ (Hazard Quotient, or ratio of ADRI over RfD) values are then HQ_1_ = ADRI_1_ / RfD_1_ and HQ_2_ = ADRI_2_ / RfD_2_. The HI value (Hazard Index, or sum of HQ values for risk agents with a common mode and mechanism of action) is then:

HI = HQ_1_ + HQ_2_ = ADRI_a,1_/RfD_1_ + ADRI_a,2_/RfD_2_ (2)

Assuming the two compounds act by the same mode or mechanism of action (the assumption adopted in this paper since perchlorate and other goitrogens act by the sodium-iodide symporter mechanism to produce iodide uptake inhibition into the thyroid; see [11]), Equation 2 reduces to:

HI = (ADRI_1_ + ADRI_2_)/RfD (3)

where there is now no subscript on RfD since the value is the same for all goitrogens acting through this shared mode of action, assuming ADRI has been calculated for each compound using a Toxicity Equivalency Factor or TEF to allow comparisons across different goitrogens (as is the case in this study). In perchlorate risk assessment, the TEF is called the Perchlorate Equivalent Concentration or PEC, but the principle is the same as in TEF.

The probabilistic methodology used here proceeds in the following steps:

1. The primary environmental media through which exposures occur are established; here they are water plus each food category.
2. The risk agents for which exposure data exist are established (perchlorate, nitrates and thiocyanates; other goitrogens with same mode and mechanism of action exist such as bromide and chlorate, but adequate exposure data do not yet exist, although data are being collected currently).
3. Inter-subject variability in the values of C for each of the three risk agents in water and different food categories is established as a lognormal probability density function (PDF) with median, GSD and level of truncation; these values of C use the PEC concept mentioned previously.
4. Inter-subject variability in the values of EF for each environmental medium is established as a lognormal probability density function (PDF) with median, GSD and level of truncation.
5. A random value is drawn from each of the two distributions (C and EF) for each of the three risk agents (perchlorate, nitrates and thiocyanates) and multiplied to obtain the three values of the ADRI in Equation 1. Here it is important to bear in mind the earlier caveat on averaging likely to be present for food consumption.
6. These three ADRI values are summed to obtain the total (PEC) value of the ADRI across all routes of exposure and goitrogens.
7. The process is repeated over 50,000 samples. The sampling size was determined by sequentially increasing the number of runs until stability at the upper 95% estimate of the inter-subject variability distribution was obtained (*i.e.*, the estimate of the 95th percentile value changed by less than 1%).
8. The resulting 50,000 values are summarised as a new probability density function for the ADRIs (converted into PEC) in the exposed population, which will also be lognormal since the product of lognormally distributed quantities is also lognormal.
9. An RfD is selected as the potential basis for a regulatory limit on total goitrogen intake, and the (PEC) ADRI values are converted first to Hazard Quotient (HQ) values by dividing the ADRI by the RfD for that route of exposure and then summing these across routes of exposure and gotirogens to produce an estimate of the Hazard Index (HI) values. The inter-subject variability distribution of these HI values is then used to calculate the percentage of the population with an HI value above 1 (a value of 1 or greater being considered in regulatory decisions as requiring consideration of mitigation).

These steps are repeated for each of several representative ages to characterise the age dependence of the risk results. The ages selected correspond to those available in the EPA Exposure Factors Handbook [12]: 1–3 months; 2–3 years; 6–11 years; 18–21 years; >21 years (full adult). In addition, a separate category of Pregnant Woman is included since this represents a potentially sensitive subpopulation.

The calculations of the percentage of the population with HI values exceeding 1 are performed using two different estimates of the relevant RfD, taken from Approaches 3 and 4 of the Introduction (Approaches 1 and 2 do not lend themselves to cumulative and aggregate risk assessment), as well as the two approaches to dealing with serum half-life. For these two approaches, the relevant ADRI threshold values (and hence RfDs) are:

- Approach 3: 13.5 µg/kg-day (without serum half-life correction) or 22.9 µg/kg-day (with serum half-life correction)
- Approach 4: 29.5 µg/kg-day (without serum half-life correction) or 38.9 µg/kg-day (with serum half-life correction.

In the previous paper [3] a 50% iodide uptake inhibition was identified as the critical level of inhibition above which adverse effects occur. This level of required inhibition is not fully established scientifically, however as being protective of public health in sensitive subpopulations. If instead one considers ANY level of inhibition to be significant (*i.e*., restricts exposures to those that produce 0% increase in iodide uptake inhibition, or 5% as selected by California’s OEHHA [13],the values of Approach 4 would be [3]:

- Approach 4b: 14.5 µg/kg-day (without serum half-life correction) or 23.9 µg/kg-day (with serum half-life correction)

Finally, the analysis includes a Contribution to Variance (CV) calculation, which provides an estimate of the fraction of the total variation in the risk metric (here, HI) between sampled individuals that is caused by any given factor used in the calculation. The CV is used here to identify the major contributing factors to the calculated percentage of individuals with an HI above 1. The reasoning behind the calculation is that if the variance of HI in the exposed population is caused primarily by variance in environmental concentration, in contrast to exposure factors, then the appropriate risk management strategy is to focus resources onto the task of identifying and mitigating the most significant sources of the elevated concentrations in this most highly exposed segment of the population rather than through national exposure limits for water and food and for each goitrogen separately.

For the product of two lognormally distributed quantities, the median of the distribution of the products is equal to the product of the medians of the separate distributions. There is also a simple relationship between the GSDs of the two distributions and the GSD of the distribution of the products [14].

However, the current study considers also the summation of the distributions for the different exposure pathways (water and food) and risk agents (perchlorate, nitrates and thiocyanates). For each sampled individual in the exposed population, this produces six values that are each lognormally distributed (water-perchlorate; water-nitrate; water-thiocyanates; food-perchlorate; food-nitrate; food-thiocyanates). In each case, a statistical test for lognormality was conducted using the Anderson-Darling test [15], and lognormality confirmed (including in the tails of the distribution out to three GSDs). The distribution of the sums of products of lognormally distributed quantities is not as straightforward computationally. As a result, the probabilistic risk assessment and the CV calculations here are performed within the CrystalBall software (a Microsoft Excel add-in by Oracle corporation), and the median, GSD and percentiles of the composite distribution of HI values obtained from the output of the software rather than calculated analytically for each separate age category.

## 3. Data Used

The exposure factors are taken from the most recent Exposure Factors Handbook [12]. The distributional data contained in the Handbook were fit with lognormal distribution functions (median and GSD), and then lognormality into the tails assumed based on the tests of lognormality. This allows the distributions to be estimated out to three GSDs into the ‘tails’ for the population, after which the distributions are truncated as mentioned previously. These tails are where values of ADRI will be highest on average and hence contribute most to the calculation of the percentage of people with HI values above 1.

Two individuals can have not only a different total intake rate of a food category (fruits, vegetables, meats, seafood), but different intake rates across the different food products within a category (for example, apples versus pears within the category of fruit). The exposure factors data available do not allow the latter form of differentiation. Therefore, the analysis proceeds using a typical “foodbasket” that is representative of the mix of food product intakes in the U.S. population modelled. Each individual has the same relative values of these intakes of food categories, but the total mass varies between individuals in accordance with the exposure factors data. This simplification is necessary due to the nature of the exposure factors data in which correlations between food categories are not available.

For the mean concentrations of perchlorate, nitrates and thiocyanates in water and food (sampling reports contain means rather than medians), the values are taken from the U.S. tables of the first paper [3]. The data for nitrates and thiocyanates in food are from the USDA Continuing Survey of Food Intakes by Individuals (CSFII), updated in 2010 (Data in the form of CSV files were obtained through http://www.ers.usda.gov/data-products/commodity-consumption-by-population-characteristics/documentation.aspx). The statistical test cited previously show the data are approximated well by a lognormal distribution across samples for each food category. For water, the distributional data are taken from the 2005–2006 U.S. Food and Drug Administration’s Total Dietary Study [16], normalised to the mean values of data reported by Blount *et al.* [17] for perchlorate and De Groef *et al.* [11] for nitrates (they base their U.S. dietary intakes on the report of Bruce *et al.* [18]). Again, the data are approximated as lognormal, in this case with a GSD of 1.5 as that is the measured GSD for the other goitrogen distributions.

Endogenous nitrate production based on exogenous intake in food and water is taken from the NRC [19]. Since inter-subject distributional data are not available, the same mean endogenous production per unit exogenous intake is applied to each individual.

In addition to the three compounds considered here, there are several other goitrogens with the same mode of action present in water and food. For example, chlorate is also present in measurable quantities, as are bromides. The data on these are insufficient to include in the present analysis. However, if these other compounds were included in the present analysis, they would need to be included in both the re-interpretation of the results from the Greer *et al.* [4] study and in the Monte Carlo analysis to calculate HI values. Inclusion of these compounds in the re-analysis would further increase the calculated PEC required to produce the adverse effects in the Greer *et al.* population, with the same (increased) intakes then being carried through to the Monte Carlo analysis. This would produce compensatory effects on the analysis, although the degree of compensation cannot be determined at present.

## 4. Results

As described previously, a random sample of 50,000 runs was collected under the Monte Carlo methodology. Table 1 shows the percentile values for ADRI (PEC) for the age groups in the population. All ADRI values are in units of µg/kg-day (PEC values). Again the reader should bear in mind the earlier caveat that the assumption of uncorrelated intakes of the goitrogens in food day-to-day for an individual means the percentage of individuals in the “tails” of Table 1 will be over-stated, although the degree of over-statement cannot be estimated at present given existing data. This will have the effect of producing an (unquantified) margin of safety or level of precaution into the analysis. Results are presented below in table rather than graphical form to allow for ease in obtaining accurate numerical values that might form the basis of regulatory and risk management decisions; only a single significant digit is provided to avoid over-interpretation of the results.

**Table 1:** Average Daily Rate of Intake (ADRI) values associated with the indicated percentiles of the inter-subject probability density functions (PDFs) as shown in the top row. All values are in units of µg/kg-day Perchlorate Equivalent Concentration (PEC). Separate values are provided by age groups. The upper table contains the results without serum half-life correction, and the lower table presents the same results with serum half-life correction.

**Age Group**	**Percentiles of ADRI**								
	1	5	10	30	50	70	90	95	99
1–3 months	3.2	3.5	4.7	8.2	11.2	15.2	19.9	23.4	25.7
2–3 years	3.8	4.2	5.6	9.8	13.5	18.3	23.9	28.1	30.9
6–11 years	3.8	4.2	5.6	9.8	13.5	18.3	23.9	28.1	30.9
18–21 years	2.9	3.2	4.2	7.4	10.1	13.7	17.9	21.1	23.2
>21 years	2.9	3.2	4.2	7.4	10.1	13.7	17.9	21.1	23.2
Pregnant women	3.5	3.9	5.1	9.0	12.4	16.7	21.9	25.7	28.3
**Age Group**	**Percentiles of ADRI**								
	1	5	10	30	50	70	90	95	99
1–3 months	5.4	6.0	8.0	13.9	19.1	25.8	33.8	39.8	43.7
2–3 years	6.5	7.2	9.5	16.7	22.9	31.0	40.6	47.7	52.5
6–11 years	6.5	7.2	9.5	16.7	22.9	31.0	40.6	47.7	52.5
18–21 years	4.9	5.4	7.2	12.5	17.2	23.3	30.4	35.8	39.4
>21 years	4.9	5.4	7.2	12.5	17.2	23.3	30.4	35.8	39.4
Pregnant women	6.0	6.6	8.7	15.3	21.0	28.4	37.2	43.7	48.1

The PDFs associated with this table were then used to calculate the percentage of the population, in each age group, with HI values exceeding 1. Separate values are provided for Approaches 3, 4 and 4b as defined previously (bear in mind that they represent different RfDs associated with adverse effects). These percentages are shown in Table 2.

**Table 2:** Percentages of the population in each age group that exceeds a Hazard Index (HI) of 1 under existing concentrations of perchlorate + nitrates + thiocyanates in water and food. Reference Dose (RfD) values used are as reported in Section 2. The upper table contains the results without half-life correction, and the lower table presents the same results with half-life correction.

**Age Group**	**Percentage of Population with HI > 1**					
	Approach 3		Approach 4		Approach 4b	
1–3 months	31.6		0.5		30.1	
2–3 years	50.0		1.1		46.2	
6–11 years	50.0		1.1		46.2	
18–21 years	30.4		0.3		28.2	
>21 years	30.4		0.3		28.2	
Pregnant women	40.8		0.5		37.4	
**Age Group**	**Percentage of Population with HI > 1**					
	Approach 3		Approach 4		Approach 4b	
1–3 months	32.0		5.1		30.6	
2–3 years	50.0		11.0		47.2	
6–11 years	50.0		11.0		47.2	
18–21 years	30.2		1.1		28.1	
>21 years	30.2		1.1		28.1	
Pregnant women	40.1		5.4		36.1	

Note that for Approach 3 all entries in Table 2 are close to 50%. This is a consequence of the background exposures in the Greer *et al.* [4] study being the mean for the adult population in Table 2. The values associated with Approach 4 are all 1.1% or less without serum half-life correction and 11% or less with serum half-life correction; for Approach 4b, the values are all 46.2% or less without serum half-life correction and 47.2% or less with serum half-life correction.

A Contribution to Variance calculation was conducted to determine the relative contributions of the following factors to the dispersion or variance of the inter-subject variability distributions. This is equivalent to the calculation of the contribution of each factor to the percentage of population with an HI value exceeding 1. The factors considered are:

- Perchlorate concentration in water;
- Perchlorate concentration in food;
- Nitrates concentration in water;
- Nitrates concentration in food;
- Thiocyanates concentration in water (negligible in the current study);
- Thiocyanates concentration in food;
- Water exposure factor;
- Food exposure factor.

Results are shown in Table 3 below, with and without serum half-life correction.

Two important results emerge from Table 3. First, the contribution to variance is dominated by nitrates concentration in food without serum half-life correction, but by both nitrates and thiocyanates in food with serum half-life correction. Second, the contribution to variance by the exposure factors is only 10–15% of the total variance. The former result indicates that regulatory focus is most appropriately on nitrates in food (without serum half-life correction) or both nitrates and thiocyanates in food (with serum half-life correction) with risk reduction focused firstly on reducing these concentrations as a priority in protection of public health.

Finally, the Monte Carlo analysis was repeated with a slight change in the question addressed. In this instance, the percentage of the population for which the HI value is above 1 is assessed for each individual goitrogen; *i.e*. the percentage for whom exposure to this goitrogen alone would produce a value of HI above 1. Results are provided in Table 4 below.

Table 4 can also be sub-divided into the contributions from water and food pathways. To obtain the percentages associated with perchlorate in water, multiply the total perchlorate contribution under any of the three approaches (with or without serum half-life correction) by 4%. For nitrates in water, multiply the total nitrates contribution under any of the three approaches (with or without serum half-life correction) by 6%. For thiocyanates, the contribution is solely from food in this study.

## 5. Conclusions

This study uses iodide uptake inhibition as a marker for adverse effect, the regulatory aim being to prevent such adverse effects. While uptake inhibition is not strictly an adverse effect in and of itself, it is at the least one mechanism that will cascade upwards to adverse effects if the perturbation to thyroid function is sufficient. Hence the use of the precursor (uptake inhibition) rather than data on explicitly adverse effects, provides a degree of conservatism or precaution into the analysis.

For Approach 3, the percentages of individuals with an HI above 1 ranges between 30.2% and 50% both with and without serum half-life correction. This is because any increase in background goitrogen exposures assumed for the Greer *et al.* study [4] produces simultaneously a higher LOEL (PEC) and higher ADRIs for Table 1. As a result, the mean of the ADRI values equals the mean of the background exposures assigned in interpreting the Greer *et al.* [4] study.

The same is not true for Approach 4 due to the use of the exposure-response function instead of a LOEL. In this case, for a 50% required inhibition, the percentage of the population with an HI above 1 is 1.1% or less with no serum half-life correction, rising to as high as 11% for some age groups when serum half-life correction is applied.

For Approach 4b, which uses a threshold of 0% inhibition, the percentage of the population with an HI above 1 is 46.2% or less with no serum half-life correction, rising to as high as 47.2% for some age groups when serum half-life correction is applied

**Table 3:** Contribution to Variance results, showing the percentage of the inter-subject variation in ADRI associated with each of the indicated factors (columns) associated with each age group. The upper table contains the results without half-life correction, and the lower table presents the same results with half-life correction.

**Age Group**	**Contribution to Varianc**								
	**Perchlorate in Water**	**Perchlorate in Food**	**Nitrate in Water**	**Nitrate in Food**	**Thiocyanate in Water**	**Thiocyanate in Food**	**Water Exposure Factor**	**Food Exposure Factor**	**SUM**
1–3 months	2	3	17	49	0	14	4	11	100
2–3 years	3	5	13	52	0	15	4	8	100
6–11 years	3	5	14	55	0	13	3	7	100
18–21 years	3	4	15	56	0	12	3	7	100
>21 years	2	4	15	57	0	12	3	7	100
Pregnant women	2	4	15	58	0	11	3	7	100
Contribution to variance									
**Age Group**	**Perchlorate in Water**	**Perchlorate in Food**	**Nitrate in Water**	**Nitrate in Food**	**Thiocyanate in Water**	**Thiocyanate in Food**	**Water Exposure Factor**	**Food Exposure Factor**	**SUM**
1–3 months	2	3	8	19	0	53	4	11	100
2–3 years	3	5	7	18	0	55	4	8	100
6–11 years	3	5	7	22	0	53	3	7	100
18–21 years	3	4	6	20	0	57	3	7	100
>21 years	2	4	6	20	0	58	3	7	100
Pregnant women	2	4	9	23	0	52	3	7	100

**Table 4:** Summary of results for the percentage of the total population with an HI value above 1, solely from the intakes of the individual goitrogens examined. The sums of these percentages are the values in Table 2, as Table 2 does not sub-divide results by goitrogen. The upper table contains the results without half-life correction, and the lower table presents the same results with half-life correction.

**Age Group**	**Percentage of Population with HI > 1 Due to the Single Goitrogen Indicated**								
	Approach 3			Approach 4			Approach 4b		
	Perchlorate	Nitrates	Thiocyanates	Perchlorate	Nitrates	Thiocyanates	Perchlorate	Nitrates	Thiocyanates
									
1–3 months	0.25	28.20	3.10	0.00	0.42	0.08	0.22	26.78	3.10
2–3 years	0.40	46.50	3.13	0.01	0.81	0.25	0.38	43.60	2.22
6–11 years	0.35	47.30	2.30	0.01	0.89	0.20	0.31	43.67	2.22
18–21 years	0.21	29.10	1.07	0.00	0.24	0.06	0.18	26.90	1.12
>21 years	0.24	28.90	1.26	0.00	0.20	0.05	0.18	26.90	1.12
Pregnant women	0.29	38.40	2.10	0.00	0.39	0.11	0.24	35.10	2.06
**Age Group**	**Percentage of Population with HI > 1 Due to the Single Goitrogen Indicated**								
	Approach 3			Approach 4			Approach 4b		
	Perchlorate	Nitrates	Thiocyanates	Perchlorate	Nitrates	Thiocyanates	Perchlorate	Nitrates	Thiocyanates
									
1–3 months	0.25	9.70	22.00	0.00	1.50	3.60	0.22	9.30	21.08
2–3 years	0.40	9.60	40.00	0.01	3.19	7.80	0.38	9.60	37.22
6–11 years	0.35	11.50	38.10	0.01	3.19	7.80	0.31	10.10	36.79
18–21 years	0.21	9.20	20.80	0.00	0.28	0.82	0.18	8.85	19.07
>21 years	0.24	9.20	20.80	0.00	0.32	0.78	0.18	8.85	19.07
Pregnant women	0.29	9.40	30.40	0.00	1.80	3.60	0.24	8.20	27.66

Several other conclusions emerge from this probabilistic approach to risk assessment for cumulative and aggregate risks from the goitrogens considered here:

- While there are exposed groups and/or sensitive subpopulations where perchlorate exposures are the primary cause of individuals having HI values above 1, these pockets constitute significantly less than 1% of the population (see Table 4).
- For individuals with an HI value above 1, the potential risk from exposure to goitrogens is dominated by nitrates when there is no serum half-life correction and thiocyanates with serum half-life correction applied (again, see Table 4).
- If perchlorate is to be regulated in water and /or food, effective allocation of risk management resources for goitrogen-induced effects is best focused on identifying the pockets of the exposed population and/or sensitive subpopulations in which perchlorate intakes through water and/or food are the cause of individuals having HI values exceeding 1 (see Table 4). The analysis suggests this will be the case in less than 0.4% of the population for water and food together in the US population examined here.

Finally, it should be noted that the Monte Carlo analysis employed here contains the implicit assumption that a sampled individual is exposed at the same concentration to a given goitrogen in water or a food category each day. For example, if they are selected (from the probability distributions) to consume water at a perchlorate concentration of X, they will have this same concentration in each day. This in turn assumes perfect correlation between the daily concentrations to which an individual is exposed over any multi-day period of time, for a given exposure pathway and water/food category.

This assumption may be valid for water intakes, since individuals tend to receive water from a single source throughout an exposure period (again, unless they are travelling). However for food, an individual will consume different mixtures of food products as well food obtained from different sources for the same food product over a period of time. When considering acute disease, this issue is not significant, since one is interested in individual daily exposures within the risk assessment. However, for short term effects such as the 14 day study characteristic of the Greer *et al.* [4] study or chronic exposures, the assumption of perfect correlation between daily concentrations will produce a wider (higher variance) distribution of exposures between individuals than would be the case if there was no correlation at all, since the sample of different sources of the food categories would tend to average out exposures to a given individual over the exposure period, moving all individuals closer to the median for the population.

The consequence of this is that the methodology used here will tend to overstate the percentage of the population with an HI value above 1 when short term or chronic exposures are of interest. Hence the current results produce an overstatement of the risk resulting from this assumption of complete correlation between daily concentrations. Unfortunately, the available data do not allow for calculation of this temporal correlation, or of its effect on results such as those in Table 1, Table 2 or Table 4. The justification for continuing with the assumption used here is that it tends to be health protective, overstating rather than understating the percentage of the population with an HI value above 1.
